# Phenotyping Bronchiectasis Frequent Exacerbator: A Single Centre Retrospective Cluster Analysis

**DOI:** 10.3390/biomedicines13092124

**Published:** 2025-08-30

**Authors:** Francesco Rocco Bertuccio, Nicola Baio, Simone Montini, Valentina Ferroni, Vittorio Chino, Lucrezia Pisanu, Marianna Russo, Ilaria Giana, Elisabetta Gallo, Lorenzo Arlando, Klodjana Mucaj, Mitela Tafa, Maria Arminio, Emanuela De Stefano, Alessandro Cascina, Amelia Grosso, Erica Gini, Federica Albicini, Virginia Valeria Ferretti, Eleonora Fresi, Angelo Guido Corsico, Giulia Maria Stella, Valentina Conio

**Affiliations:** 1 Unit of Respiratory Disease, Cardiothoracic and Vascular Department, IRCCS Policlinico San Matteo, Viale Golgi 19, 27100 Pavia, Italy; francesco.bertuccio01@gmail.com (F.R.B.); simone.montini01@universitadipavia.it (S.M.); valentina.ferroni01@universitadipavia.it (V.F.); lucrezia.pisanu01@universitadipavia.it (L.P.); marianna.russo01@universitadipavia.it (M.R.); ilaria.giana01@universitadipavia.it (I.G.); elisabetta.gallo01@universitadipavia.it (E.G.); lorenzo.arlando01@universitadipavia.it (L.A.); klodjana.mukaj01@universitadipavia.it (K.M.); mitela.tafa01@universitadipavia.it (M.T.); maria.arminio01@universitadipavia.it (M.A.); emanuela.destefano01@universitadipavia.it (E.D.S.); a.cascina@smatteo.pv.it (A.C.); amelia.grosso@gmail.com (A.G.); erica.gini@gmail.com (E.G.); federica.albicini@gmail.com (F.A.); a.corsico@smatteo.pv.it (A.G.C.); 2 Department of Internal Medicine and Medical Therapeutics, University of Pavia, 27100 Pavia, Italy; 3 Ospedale Maggiore, ASST Crema, 26013 Crema, Italy; nicola.baio01@universitadipavia.it; 4 Ospedale Pederzoli, Peschiera del Garda, 37121 Verona, Italy; vittorio.chino01@universitadipavia.it; 5 Biostatistica e Clinical Trial Center, Direzione Scientifica, Fondazione IRCCS Policlinico San Matteo, Viale Golgi 19, 27100 Pavia, Italy; v.ferretti@smatteo.pv.it (V.V.F.); e.fresi@smatteo.pv.it (E.F.)

**Keywords:** non-cystic fibrosis bronchiectasis, phenotypic clusters, multivariate analyses, clinical outcomes

## Abstract

**Background:** Bronchiectasis is a chronic respiratory condition characterized by permanent bronchial dilation, recurrent infections, and progressive lung damage. A subset of patients, known as frequent exacerbators, experience multiple exacerbations annually, leading to accelerated lung function decline, hospitalizations, and reduced quality of life. The aim of this study is to identify distinct phenotypes and treatable traits in bronchiectasis frequent exacerbators, since it could be crucial for optimizing patient management. **Research question:** Could clinically distinct phenotypes and treatable traits be identified among frequent exacerbators with bronchiectasis to guide personalized management strategies? **Methods:** We analysed a cohort of 56 bronchiectasis frequent exacerbator patients using 21 clinically relevant variables, including pulmonary function tests, radiological patterns, and microbiological data. Hierarchical clustering and k-means algorithms were applied to identify subgroups. Key outcomes included cluster-specific characteristics, treatable traits, and their implications for management. **Results:** Four distinct clusters were identified: 1. Mild, idiopathic bronchiectasis (Cluster 1): Predominantly mild disease (FACED), idiopathic etiology (93.3%), and cylindrical bronchiectasis with moderate obstruction (60%). 2. Rheumatological and NTM-associated bronchiectasis (Cluster 2): Patients with systemic inflammatory diseases (50%) and NTMever (50%) but minimal infections by *Pseudomonas aeruginosa*. 3. Mild, post-infective bronchiectasis (Cluster 3): Exclusively mild disease, mixed idiopathic and post-infective etiologies, and preserved lung function. 4. Severe, chronic infection phenotype (Cluster 4): Severe disease with high colonization rates of *Pseudomonas aeruginosa* (71.4%), advanced structural damage (57.1% varicose, 50% cystic bronchiectasis), and frequent exacerbations. **Interpretation:** This analysis highlights the heterogeneity of bronchiectasis and its frequent exacerbator phenotype. The treatable traits framework underscores the importance of aggressive infection control and management of airway inflammation in severe cases, while milder clusters may benefit from preventive strategies. These findings support the integration of precision medicine in bronchiectasis care, focusing on phenotype-specific interventions to improve outcomes.

## 1. Introduction

Bronchiectasis is a chronic and heterogeneous respiratory condition characterized by irreversible bronchial dilation, recurrent infections, and progressive lung damage. Patients frequently present with a wide range of clinical manifestations, from mild disease with minimal symptoms to severe cases complicated by frequent exacerbations, chronic infections, and systemic comorbidities [1]. Among them, the frequent exacerbator phenotype is particularly concerning, as these patients experience multiple exacerbations per year, leading to rapid lung function decline, reduced quality of life, and increased mortality [2]. Identifying and managing this phenotype is crucial to improving outcomes and reducing the disease burden. There are no approved treatments for bronchiectasis, and the evidence supporting widely used approaches like physiotherapy and long-term macrolide therapy is insufficient. Consequently, an individualised therapeutic approach could be effective in disease management [3].

Cluster analysis, a statistical method for grouping individuals based on shared characteristics, has emerged as a powerful tool to better understand the heterogeneity of bronchiectasis. By identifying distinct phenotypes within the broader disease population, this approach enables targeted treatment strategies and the identification of underlying disease mechanisms [4].

Phenotypes commonly identified through clustering include those defined by infection profiles, radiological patterns, and disease severity, reflecting the complex interplay of factors driving disease progression [4].

Furthermore, the concept of treatable traits enhances this precision-based approach. Treatable traits are specific, modifiable features (such as chronic infections, airway inflammation, and comorbidities) that can guide personalized therapy. In bronchiectasis, traits like *Pseudomonas aeruginosa* colonization, severe airflow obstruction, and systemic conditions such as gastroesophageal reflux disease (GERD) or rheumatological diseases are increasingly recognized as potential targets for intervention [3,5].

Combining cluster analysis with a treatable traits framework provides an opportunity to refine phenotyping and prioritize actionable clinical characteristics [3].

Our study integrates cluster analysis and treatable trait identification to explore the heterogeneity of bronchiectasis, with a particular focus on the frequent exacerbator phenotype. By characterizing clinical, radiological, and microbiological profiles across a cohort of patients, it aims to provide insights into distinct phenotypes, enabling more effective, personalized management of this challenging condition.

## 2. Materials and Methods

### 2.1. Study Design and Objective

This retrospective study analysed a cohort of 56 patients with non-cystic fibrosis bronchiectasis, frequent exacerbator phenotype, focusing on clinically significant variables. This study was approved by the local ethical committee. Patients were included if they fulfilled a frequent exacerbator phenotype, defined as having ≥ 3 exacerbations in the preceding 12 months.

The primary objective of this study was to stratify frequent exacerbators into distinct subgroups based on clinical, radiological, and microbiological characteristics, enabling a deeper understanding of disease heterogeneity and informing personalized treatment strategies. The findings highlight key variables that serve as predictors of disease progression and unfavorable outcomes, allowing for refined risk stratification.

### 2.2. Study Population

Patients were recruited based on established clinical and radiological criteria and referred to our bronchiectasis clinics of university teaching hospitals in Pavia (Italy). Patients with cystic fibrosis or traction bronchiectasis due to pulmonary fibrosis were excluded.

We utilise the following consensus definition for exacerbations established in 2017 by an international group of experts on bronchiectasis for clinical trials: “deterioration of three or more key symptoms for at least 48 h, in addition to a clinician’s decision that a change in bronchiectasis treatment. The key symptoms are (1) cough, (2) sputum volume and/or consistency, (3) sputum purulence, (4) breathlessness and/or exercise intolerance, (5) fatigue and/or malaise, and (6) haemoptysis” [6].

Moreover, we adopted the currently approved definition of “frequent exacerbator” in bronchiectasis as a patient who experiences three or more exacerbations per year, according to the literature [2].

This phenotype is associated with worse clinical outcomes, including accelerated lung function decline, impaired quality of life, and increased healthcare utilization [2].

At the time of clinical assessment, all patients underwent the diagnostic work-up suggested by the 2010 British Thoracic Society (BTS) guidelines [7].

An exacerbation was counted if it was (i) documented in the electronic health record (EHR) as a physician-confirmed acute deterioration requiring systemic antibiotics and/or corticosteroids, or (ii) resulted in unplanned emergency department attendance or hospital admission. Patient self-reports during clinical interview were used only to cross-check EHR-derived information, not as the primary source of exacerbation ascertainment.

### 2.3. Clinical Measurements

A total of 21 clinically significant variables were analysed. Variables included demographic data (age, sex), clinical markers (FACED score, history of exacerbations and hospitalizations), pulmonary function tests (FVC, FEV1, PFT obstruction), radiological findings (cylindrical, varicose, and cystic bronchiectasis), and microbiological data (chronic colonization by *Pseudomonas aeruginosa*, Staphylococcus aureus, Nontuberculous Mycobacteria [NTM], and fungal infections). Etiology (e.g., idiopathic, post-infective, rheumatological) and systemic comorbidities (e.g., GERD, hypertension, rheumatological diseases) were also included to account for the heterogeneity of the disease.

The Charlson Comorbidity Index (CCI) was used to assess comorbidities [8]. The severity of bronchiectasis was evaluated according to both the Bronchiectasis Severity Index (BSI) and FACED score (evaluating forced expiratory volume in 1 s, age, chronic infection with Pseudomonas, radiological extension, and dyspnoea) [9,10]. Radiological severity of bronchiectasis was assessed using a modified Reiff score, which rates the number of involved lobes and the degree of dilatation (range 1–18) [11]. All bacteriology was performed on spontaneous sputum or bronchoalveolar lavage samples. Chronic infection was defined by the isolation of potentially pathogenic bacteria in sputum culture on two or more occasions, at least 3 months apart over a 1-year period.

All patients exhibited characteristic features of the disease, including permanent bronchial dilation confirmed via high-resolution computed tomography (HRCT), as well as chronic respiratory symptoms such as productive cough, dyspnea, and recurrent exacerbations [12].

### 2.4. Data Collection

A total of 21 baseline clinical, functional, radiological, and microbiological variables were extracted from the EHR and verified against the prospectively maintained bronchiectasis database. Collected data included:

- Demographics (age, sex, body mass index).
- Lung function (FEV_1_% predicted, FVC% predicted, FEV_1_/FVC ratio).
- Radiology (extent and morphology of bronchiectasis, Reiff score).
- Clinical scores (FACED, BSI).
- Comorbidities (e.g., rheumatoid arthritis, gastroesophageal reflux).
- Microbiology (chronic colonisation with *Pseudomonas aeruginosa* or other pathogens, NTM-ever).

Where missing data were present (<10% for all variables), we applied single imputation: median for continuous variables and mode for categorical variables.

### 2.5. Data Analysis

All analyses were conducted using R (v. 4.3.1). We performed patient stratification through cluster analysis, leveraging longitudinal data from frequent exacerbators who have been under continuous follow-up at our centre since their initial presentation.

**Figure 1:**
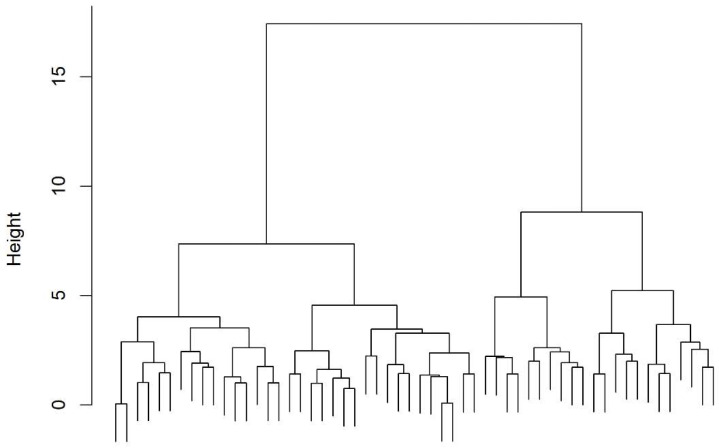
Dendrogram for Hierarchical Clustering with Ward distance.

Hierarchical clustering using Ward’s method was first applied to explore the dataset structure and determine the optimal number of clusters (Figure 1). This approach grouped patients based on Euclidean distances while minimizing within-cluster variance. Cluster quality was assessed using the silhouette coefficient and Dunn index. Based on these results, k-means clustering was then performed.

Cluster-specific characteristics were analyzed using descriptive statistics and compared using Fisher’s exact test for categorical variables and the Kruskal-Wallis test for continuous variables. Variables with statistically significant differences (*p* < 0.05) were highlighted to identify distinguishing traits of each cluster. The results of the clustering processes were reported graphically as dendrograms and cluster plots.

### 2.6. Data Preprocessing

- Continuous variables were z-standardised.
- Categorical variables were one-hot encoded prior to clustering.
- Sensitivity analyses compared the imputed dataset with a complete-case dataset; cluster allocation was unchanged.

### 2.7. Clustering Procedure

Primary clustering was performed in a two-step approach:

1. Agglomerative hierarchical clustering (Ward’s linkage, Euclidean distance) applied to the standardised feature set.
2. K-means consolidation to refine cluster membership.

Given the presence of mixed data types, a sensitivity analysis using Gower distance and Partitioning Around Medoids (PAM) was conducted. The alternative approach yielded a highly concordant four-cluster solution (Adjusted Rand Index 0.82, Normalised Mutual Information 0.78) compared with the primary method.

### 2.8. Cluster Validation

Internal validity was assessed across candidate solutions (k = 2–6) using multiple indices:

- Average silhouette width: 0.14 (optimal at k = 4).
- Dunn index: 0.42.
- Gap statistic: supported k = 4.
- Elbow plot: suggested a four-cluster solution.

Cluster stability was tested by bootstrap resampling (1000 iterations). Jaccard similarity coefficients were 0.61, 0.59, 0.64, and 0.67 for clusters 1–4, respectively, indicating moderate reproducibility given the cohort size.

### 2.9. Visualization

**Figure 2:**
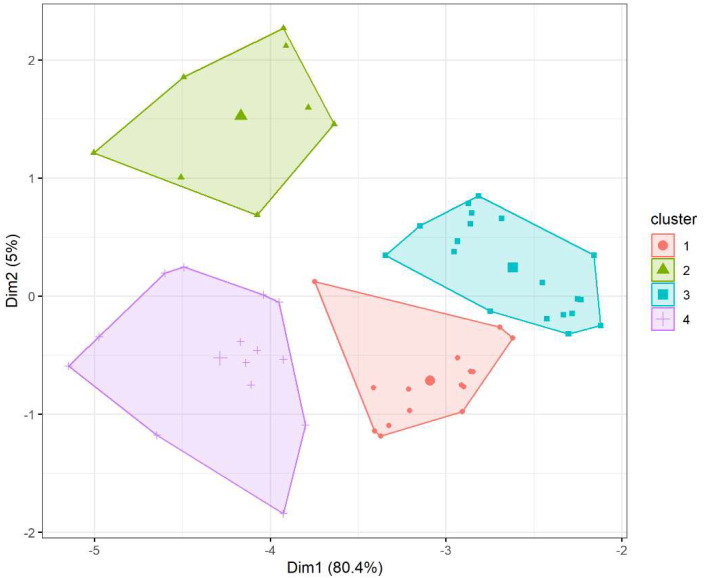
K-means clustering for patients.

For descriptive purposes, low-dimensional projections were generated using Principal Component Analysis (PCA) and Multiple Correspondence Analysis (MCA). Ellipses in Figure 2 represent 95% confidence regions calculated using the multivariate *t*-distribution method (stat_ellipse (type = “t”) in ggplot2). We emphasise that these visualisations were used only for illustration; quantitative evaluation of cluster separation relied on the full multidimensional feature space.

## 3. Results

### 3.1. Patients

Fifty-six patients diagnosed with bronchiectasis with a frequent exacerbator phenotype were involved in the analysis as stated above. Patients’ characteristics are shown in Table 1. Median age is 67 years, with a prevalence of female sex (40 vs. 16 males). The majority of patients were predominantly composed of nonsmokers. The most reported etiology was idiopathic bronchiectasis, followed by post-infectious and rheumatic disease-associated bronchiectasis. Most frequently observed comorbidities include cardiovascular diseases, GERD, and COPD.

More than half of patients had an extensive radiological presentation with three or more lobes involved. The most frequent radiologic phenotype was cylindric bronchiectasis.

Dominant symptoms include cough and sputum production. Severe dyspnea (mMRC 3–4) was observed in 24 patients. Functional obstruction was detected in 25 patients, whereas data regarding DLCO were not available.

Concerning chronic infections, *P. aeruginosa* accounted for most cases (17); other less frequent colonizations were driven by *S. aureus* and *Aspergillus* spp. NTM chronic infection affected 9 patients, sustained mainly by *M. avium*.

The majority of patients included in the study were vaccinated against S. pneumoniae and H. influenzae.

Approximately 50% of frequent exacerbators in our cohort were already performing daily physiotherapy exercises. Median symptom duration was 13 years, and a significantly higher proportion of frequent exacerbators experienced a previous hospitalization 84%. Previous bacterial isolation has been found strongly associated with frequent exacerbations, where more than 90% of patients had prior documented infection.

### 3.2. Cluster Analysis

Hierarchical clustering suggested the presence of four homogeneous clusters (Figure 1), consistent with findings in the literature. Based on this result, k-means clustering was performed, yielding a separation index of 1.42. Results of K-means clustering are visualized in Figure 2, which illustrates well-defined clustering and supports the robustness of the identified subgroups.

This cluster analysis provides a framework for applying the treatable trait model to bronchiectasis frequent exacerbators, offering actionable insights for each identified subgroup.

### 3.3. Cluster Features

The characteristics of patients, categorized according to the cluster to which they have been assigned, are presented in Table 2.

Cluster 1 (Mild, Idiopathic) (N = 15): patients exhibit predominantly mild to moderate disease severity according to the FACED score (46.7% mild, 53.3% moderate). High prevalence of idiopathic etiology (93.3%), with minimal cases of post-infective bronchiectasis, was evidenced. All patients in this cluster have cylindrical bronchiectasis without significant varicose or cystic changes. Patients show a moderate presence of pulmonary function obstruction (60%) with FVC values around 0.80 L. Concerning infections, there is a moderate colonization by *Pseudomonas aeruginosa* (33.3%). In this group, symptoms predominantly began after 2010 (58.3%), suggesting a group of relatively recent disease onset.

Cluster 2 (Reumatological and NTM-Associated) (N = 8): patients show predominantly mild disease severity (62.5% mild) with a distinct etiology pattern: 50% reumatological causes, reflecting an atypical subgroup within bronchiectasis populations. As the cluster’s name implies, there is a high prevalence of NTMever (Non-tuberculous mycobacterial infection, 50%), aligning with reports of increased Nontuberculous Mycobacteria in immune-compromised or inflammatory conditions. Most patients exhibit cylindrical bronchiectasis (87.5%), consistent with inflammatory rather than infectious origins. FVC values and obstruction levels are within the mid-range for the dataset. No patients exhibit colonization by *Pseudomonas aeruginosa*. Symptoms frequently began after 2010 (83.3%), further distinguishing this group from clusters with long-standing disease.

Cluster 3 (Mild, Post-Infective) (N = 19) This group comprises exclusively mild disease cases based on the FACED score. Etiology is mixed, with idiopathic (52.6%) and post-infective (47.4%) causes equally represented. Patients exhibit only cylindrical bronchiectasis with relatively preserved lung function. Obstruction is rare in this group (15.8%), correlating with better outcomes in the literature. A low prevalence of *Pseudomonas aeruginosa* colonization (10.5%) and mild incidence of Staphylococcus aureus infections are highlighted. Symptoms began predominantly after 2000 (57.9%), reflecting a less chronic disease course compared to Cluster 4.

Cluster 4 (Severe, Chronic, and Infected) (N = 14): represents the most severe subgroup, with equal distribution of moderate and severe FACED scores (50% each). It comprehends a high prevalence of post-infective (64.3%) and rheumatological (28.6%) causes, with an extensive disease history. This cluster shows the most advanced bronchiectatic changes: 57.1% varicose bronchiectasis, 50% cystic bronchiectasis, and the lowest FVC (0.68 ± 0.2). Severe pulmonary obstruction (78.6%) is common, aligning with descriptions of poor functional outcomes in advanced disease. Moreover, extensive colonization by *Pseudomonas aeruginosa* (71.4%) and *Aspergillus fumigatus* (42.9%), both associated with frequent exacerbations and worse quality of life, is reported. Symptoms typically began before 2000 (83.3%), reflecting a long-standing disease course. As additional features, the prevalence of GERD (71.4%), daily sputum production (92.9%), and most recurrent exacerbations are reported in this group.

## 4. Discussion

Although our study included only 56 patients from a single referral centre, the baseline characteristics of our cohort (median age 67 years, 71% female, chronic *Pseudomonas* colonisation 30%) are consistent with those reported in large registries such as EMBARC. Nevertheless, enrichment for the frequent exacerbator phenotype and our single-centre setting limit generalisability. Therefore, our findings should be regarded as hypothesis-generating and require validation in multicentre and prospective studies.

A major limitation is the absence of external validation and prospective outcome data. We did not assess whether clusters differed in future exacerbations, lung function decline, or quality-of-life trajectories. These analyses are planned in an ongoing longitudinal follow-up and will be essential to test the prognostic value of the identified phenotypes.

As an example, Cluster 2 contained only eight individuals, which raises concerns about reproducibility and overfitting. While the clinical profile was coherent, such small groups require confirmation in larger datasets. Our conclusions should therefore be interpreted as hypothesis-generating.

The analysis of our bronchiectasis cohort highlights distinct phenotypes divided into 4 clusters (Figure 2). In order to strengthen our analysis, we briefly compared each cluster with the literature and highlighted potential treatable traits.

### 4.1. Cluster 1

Similar clusters have been identified in studies grouping patients with milder disease and idiopathic bronchiectasis (Martínez-García et al., 2014), reflecting better prognosis and less aggressive disease courses [13].

#### Treatable Traits

1. Idiopathic Etiology:

These patients require thorough evaluation to rule out undiagnosed causes, such as immunodeficiencies or undetected infections.

Routine monitoring may suffice due to the mild disease burden.

2. Airway Clearance:

With moderate pulmonary obstruction (60%), these patients could benefit from airway clearance techniques to prevent exacerbations and maintain lung function.

3. Infrequent Infections:

Periodic screening for pathogens like *Pseudomonas aeruginosa* ensures timely intervention if colonization occurs.

### 4.2. Cluster 2

This group mirrors findings in clusters dominated by non-infectious or systemic disease causes (Aksamit et al., 2017), which often require tailored anti-inflammatory or immune-modulating strategies [14].

#### Treatable Traits

1. Reumatological Diseases:

The high prevalence of systemic inflammatory conditions (50% with rheumatological etiology) highlights the need for coordinated care with rheumatologists.

Targeted immunomodulatory therapy could address the underlying systemic condition and improve bronchiectasis outcomes.

2. NTM Colonization:

The presence of NTMever in 50% of patients necessitates careful microbiological surveillance and, where appropriate, long-term antimicrobial therapy to manage infections.

3. GERD:

With GERD present in 50% of patients, optimizing reflux management (e.g., proton pump inhibitors, dietary adjustments) could mitigate recurrent aspirations and bronchiectasis progression.

### 4.3. Cluster 3

This cluster aligns with reports of post-infective bronchiectasis in non-severe forms, often linked to improved prognosis if infections are controlled (Goeminne et al., 2018) [15].

#### Treatable Traits

1. Post-Infective Etiology:

A focus on preventing recurrent infections (e.g., vaccination for pneumococcus and influenza, prophylactic antibiotics if indicated) is crucial to avoid progression.

2. Good Pulmonary Function:

Preserved FVC (0.92 ± 0.2) indicates a window for preventive measures to maintain lung health, emphasizing regular follow-ups and infection control.

3. Infrequent Colonization:

Low rates of *Pseudomonas aeruginosa* (10.5%) and *S. aureus* (36.8%) suggest that these patients benefit from conservative antibiotic use unless colonization worsens.

### 4.4. Cluster 4

This cluster corresponds to the “high-risk” subgroups identified in studies on severe bronchiectasis, marked by persistent Pseudomonas colonization and extensive radiological damage (Chalmers et al., 2018, and Araujo in 2018 too) [5,16].

#### Treatable Traits

1. Chronic Infections:

High colonization rates of *Pseudomonas aeruginosa* (71.4%) and *Aspergillus fumigatus* (42.9%) warrant aggressive antimicrobial management, such as inhaled antibiotics or antifungal therapy.

Eradication attempts for Pseudomonas in early stages of colonization could prevent long-term sequelae.

2. Advanced Structural Damage:

The prevalence of varicose (57.1%) and cystic bronchiectasis (50%) highlights the importance of pulmonary rehabilitation and regular imaging to monitor progression.

3. Airway Inflammation and Obstruction:

Severe pulmonary obstruction (78.6%) suggests a need for bronchodilator therapy and optimization of anti-inflammatory treatments, potentially including inhaled corticosteroids in specific contexts.

4. GERD and Aspiration:

GERD is present in 71.4% of these patients, making reflux control critical to reducing exacerbations and preventing further lung injury.

5. Frequent Exacerbations:

Daily sputum production (92.9%) and a history of frequent infections necessitate exacerbation prevention strategies, including maintenance antibiotics and physiotherapy.

In our study, patients included in cluster 4 demonstrated high rates of *Pseudomonas aeruginosa* colonization (71.4%) and varicose or cystic bronchiectasis, consistent with these findings.

Chronic infections are a hallmark of frequent exacerbators, with *Pseudomonas aeruginosa* being a dominant pathogen. This pathogen is known to increase the risk of exacerbations, accelerate lung function decline, and correlate with greater radiological damage [5]. Our data confirm these associations, as Cluster 4 patients exhibited the worst functional impairment (mean FVC 0.68 ± 0.2) and severe airflow obstruction. Compared to literature, where exacerbations are linked to systemic inflammation, the frequent association of GERD in Cluster 4 (71.4%) emphasizes the role of aspiration in perpetuating the cycle of infection and inflammation, as previously reported by Lee et al. [17].

Interestingly, our results also identified milder phenotypes (Clusters 1 and 3) with preserved lung function, low infection burden, and predominantly idiopathic or post-infective etiologies. These findings parallel reports of stable disease phenotypes, where outcomes are better if exacerbations and infections are controlled early (Martínez-García et al., 2014) [13]. Cluster 2, dominated by rheumatological and NTM cases, highlights the importance of systemic disease as a driver of bronchiectasis phenotypes, aligning with emerging research on immune-modulated forms of the disease [18].

Overall, our findings emphasize the heterogeneity of bronchiectasis and the need for targeted interventions based on phenotyping and treatable traits. The frequent exacerbator phenotype, as identified in Cluster 4, requires aggressive management to mitigate infections, address systemic drivers, and prevent further deterioration.

The treatable traits identified in this analysis underscore the potential for personalized treatment approaches in bronchiectasis, tailored to the unique characteristics of specific patient clusters [19]. For instance, patients in Cluster 2 may benefit from therapies targeting Nontuberculous Mycobacteria (NTM), while those in Cluster 4 require aggressive infection control strategies due to the high prevalence of *Pseudomonas aeruginosa* colonization. In contrast, preventive measures aimed at reducing exacerbations and preserving lung function are crucial for Clusters 1 and 3, which are characterized by milder disease profiles.

A multidisciplinary approach is essential, particularly for Clusters 2 and 4, where systemic conditions and chronic infections intersect with lung disease. Coordinated care involving pulmonology, rheumatology, and gastroenterology can address the complex interplay of factors contributing to disease progression and optimize patient outcomes [20].

Proactive monitoring plays a key role in managing bronchiectasis across all clusters. Regular assessments of lung function, radiological changes, and microbiological colonization are critical for identifying modifiable factors early and implementing timely interventions to prevent disease worsening.

While this analysis offers robust insights, certain limitations must be addressed:

First, the relatively small sample size (N = 56) may limit the generalizability of findings. Second, overlapping features between clusters potentially indicate the need for additional variables or clustering methods.

Moreover, the retrospective nature of the study may confound data quality. Furthermore, a selection bias could be present due to the fact that our Institution is a Reference Center and that our cohort is composed mainly of patients with long-lasting disease, implying that the presence of factors associated with newly diagnosed disease could be underestimated. Finally, data regarding mortality, previous treatment history, complete pulmonary function tests, and clinical outcomes are missing, limiting the quality of results.

And lastly, the inclusion of inflammatory markers (e.g., CRP, eosinophils) and biomarkers (e.g., sputum proteomics) in future analyses may enhance cluster differentiation [21].

Beyond CT and microbiology, lung-acoustic analysis is emerging as a non-invasive complement to phenotyping. Resonance-based tunable-wavelet pipelines can separate co-existing crackles and wheezes [22,23], transformer models can grade COPD severity directly from auscultation [22,23], and self-supervised pre-training has improved crackle detection in bronchiectasis datasets [22,23]. Integration of such multimodal signals with clinical and radiological features may refine phenotype classification and enable real-time monitoring in future studies.

Furthermore, the mapping of phenotypes to treatable traits should be regarded as exploratory. Prospective studies are needed to determine whether these clusters predict differential outcomes or responses to therapy.

Overall, this analysis emphasizes the value of the treatable trait framework in driving improved outcomes through precise, individualized management strategies. By addressing the diverse needs of bronchiectasis patients, this approach holds the promise of reducing disease burden and enhancing quality of life.

## 5. Conclusions and Interpretation

This study highlights the heterogeneity of bronchiectasis, identifying distinct phenotypes through cluster analysis, including the high-risk frequent exacerbator group characterized by severe disease, advanced structural damage, and chronic *Pseudomonas aeruginosa* colonization. These findings identify treatable traits such as chronic infections and systemic comorbidities as actionable targets for personalized interventions. Future clinical trials should focus on phenotype-driven approaches, evaluating the efficacy of tailored therapies, such as long-term antibiotics, anti-inflammatory agents, and GERD management, in improving outcomes. Moreover, a better comprehension of underlying inflammatory drivers through non-invasive analysis methods, such as those used for detecting peripheral biomarkers, is of immense importance for disease management. This perspective emphasizes the potential of precision medicine to optimize care for bronchiectasis patients and reduce disease burden.

## Figures and Tables

**Table 1:** Overall Patient Characteristics.

Variable	N = 56 ^1^
**Age years**	67.00 (61.0, 74.50)
**Sex**	
F	40 (71.4%)
M	16 (28.6%)
**Year_Start_symptoms**	2010 (1985, 2017)
**Smoking**	
Never	31 (57.4%)
Active	7 (13.0%)
Ex	16 (29.6%)
**BMI** **kg/m^2^**	23.23 (20.0, 27.00)
**BSI**	
mild	6 (11.5%)
moderate	7 (13.5%)
severe	39 (75.0%)
**Cilindric**	54 (96.4%)
**Varicose**	11 (20.0%)
**Cystic**	15 (26.8%)
**Etiology**	
idiopathic	25 (46.3%)
postinfective	19 (35.2%)
rheumatic	6 (11.1%)
other	4 (7.4%)
**FEV1 %**	0.76 (0.5, 0.92)
**FVC %**	0.84 (0.7, 0.98)
**COPD**	10 (17.9%)
**Asthma**	6 (10.7%)
**Reiff_score**	
1	20 (35.7%)
2	36 (64.3%)
**Sinusitis**	7 (12.7%)
**Arterial_hypertension**	22 (39.3%)
**Previous_AMI**	2 (3.6%)
**Angina**	0 (0%)
**Previous_stroke**	1 (1.8%)
**Vasculopathy**	4 (7.1%)
**Previous_PTCSA**	2 (3.6%)
**Atrial_fibrillation**	7 (12.5%)
**Valvulopathy**	6 (10.7%)
**Chronic_heart_failure**	5 (8.9%)
**Pulmonary_hypertension**	3 (5.4%)
**Hypercholesterolemia**	10 (17.9%)
**Diabetis**	7 (12.5%)
**Liver_disease**	4 (7.1%)
**Chirrosis**	1 (1.8%)
**Chronci_renal_failure**	1 (1.8%)
**Neurological_disease**	4 (7.1%)
**Dementia**	1 (1.8%)
**Rheumatological Disease**	8 (14.3%)
**Rheumatoid Arthritis**	5 (8.9%)
**Vasculitis**	2 (3.6%)
**Osteoporosis**	9 (16.1%)
**GERD**	22 (39.3%)
**Ulcerative_colitis**	2 (3.6%)
**Immunodeficit**	1 (1.8%)
**Tumor**	11 (19.6%)
**Solid_tumor**	11 (19.6%)
**Haematological_malignancy**	0 (0%)
**Statins**	9 (16.4%)
**Ace_inhibitors**	7 (12.7%)
**Sartans**	14 (25.9%)
**Aspirin**	1 (1.8%)
**Clopidogrel**	2 (3.6%)
**Anticoagulant_therapy**	6 (10.9%)
**B_blockers**	16 (29.1%)
**PPI**	28 (50.9%)
**Vaccination_influenzae**	29 (67.4%)
**Vaccination_antipneumococcal**	31 (70.5%)
**antisarscov2**	
0	2 (5.0%)
1	3 (7.5%)
2	35 (87.5%)
**Cough**	40 (72.7%)
**Sputum**	46 (83.6%)
**Sputum_daily**	35 (63.6%)
**Haemoptysis**	18 (32.7%)
**Frequency**	
Never	34 (64.2%)
Previous	9 (17.0%)
Periodic	9 (17.0%)
Daily	1 (1.9%)
**Dyspnea**	27 (48.2%)
**mMRC**	
0	29 (51.8%)
1	3 (5.4%)
2	9 (16.1%)
3	11 (19.6%)
4	4 (7.1%)
**Weight_loss**	6 (10.7%)
**Astenia**	8 (14.3%)
**Previous pneumonia**	21 (38.2%)
**Previous_Bacteria lfungal isolation**	51 (91.1%)
***Moraxella catharralis***	1 (1.8%)
***Streptococcus pneumoniae***	4 (7.1%)
***Haemophilus influenzae***	11 (19.6%)
***Staphylococcus aureus***	16 (28.6%)
***Rothia mucilaginosa***	1 (1.8%)
***Aspergillus fumigatus***	12 (21.4%)
***Aspergillus terreus***	2 (3.6%)
***Aspergillus niger***	5 (8.9%)
***Aspergillus flavus***	1 (1.8%)
***Klebsiella* spp.**	3 (5.4%)
***Nocardia* spp.**	6 (10.7%)
***Escherichia coli***	3 (5.4%)
***Enterobacter* spp.**	2 (3.6%)
***Stenotrophomonas maltophila***	1 (1.8%)
***Achromobacter* spp.**	4 (7.1%)
***Serratia marcescens***	2 (3.6%)
***Acinetobacter baumanni***	1 (1.8%)
**Previous *Pseudomonas***	30 (53.6%)
**NTM ever**	14 (25.0%)
***M. avium***	10 (17.9%)
***M. intracellulare***	2 (3.6%)
***M. abscessus***	3 (5.4%)
***M. xenopi***	1 (1.8%)
***M. fortuitum***	1 (1.8%)
***M. gordonae***	1 (1.8%)
**Previous_TB**	2 (3.6%)
**Previous_Virus**	14 (25.0%)
**Respiratory FKT**	29 (52.7%)
**Colonized**	30 (53.6%)
**Previous hospital**	47 (83.9%)

^1^ Median (Q1, Q3); n (%).

**Table 2:** (A) Patient’s clinical characteristics by clusters. (B) Patients’ symptoms and relative frequency, drug history by clusters. (C) Patients’ microbiological characteristics by clusters.

(A)					
**Variable**	**1 N = 15 ^1^**	**2 N = 8 ^1^**	**3 N = 19 ^1^**	**4 N = 14 ^1^**	***p*-Value ^2^**
**Age years**	67.00 (48.0, 73.00)	65.00 (58.5, 72.00)	64.00 (61.0, 73.00)	75.00 (64.0, 79.00)	0.10
**Sex**					0.6
Female	12 (80.0%)	6 (75.0%)	14 (73.7%)	8 (57.1%)	
Male	3 (20.0%)	2 (25.0%)	5 (26.3%)	6 (42.9%)	
**Year_Start_symptoms**					<0.001
Before 2000	5 (41.7%)	1 (16.7%)	3 (15.8%)	10 (83.3%)	
2000–2010	0 (0.0%)	0 (0.0%)	5 (26.3%)	1 (8.3%)	
After 2010	7 (58.3%)	5 (83.3%)	11 (57.9%)	1 (8.3%)	
**Smoking**					>0.9
active	2 (13.3%)	0 (0.0%)	3 (16.7%)	2 (15.4%)	
ex	5 (33.3%)	2 (25.0%)	6 (33.3%)	3 (23.1%)	
never	8 (53.3%)	6 (75.0%)	9 (50.0%)	8 (61.5%)	
**BMI kg/m^2^**	23.53 (21.8, 26.81)	26.21 (23.2, 26.81)	20.32 (19.4, 22.76)	24.69 (19.2, 29.55)	0.10
**BSI**					0.056
mild	1 (7.7%)	2 (25.0%)	3 (17.6%)	0 (0.0%)	
moderate	1 (7.7%)	2 (25.0%)	4 (23.5%)	0 (0.0%)	
severe	11 (84.6%)	4 (50.0%)	10 (58.8%)	14 (100.0%)	
**COPD**	2 (13.3%)	1 (12.5%)	3 (15.8%)	4 (28.6%)	0.8
**Asthma**	1 (6.7%)	2 (25.0%)	2 (10.5%)	1 (7.1%)	0.6
**Sinusitis**	2 (13.3%)	1 (12.5%)	2 (11.1%)	2 (14.3%)	>0.9
**Arterial_hypertension**	4 (26.7%)	2 (25.0%)	8 (42.1%)	8 (57.1%)	0.3
**Previous_AMI**	2 (13.3%)	0 (0.0%)	0 (0.0%)	0 (0.0%)	0.15
**Previous_stroke**	0 (0.0%)	1 (12.5%)	0 (0.0%)	0 (0.0%)	0.14
**Vasculopathy**	2 (13.3%)	0 (0.0%)	1 (5.3%)	1 (7.1%)	0.8
**Previous_PTCSA**	2 (13.3%)	0 (0.0%)	0 (0.0%)	0 (0.0%)	0.15
**Atrial_fibrillation**	1 (6.7%)	2 (25.0%)	1 (5.3%)	3 (21.4%)	0.3
**Valvulopathy**	2 (13.3%)	0 (0.0%)	2 (10.5%)	2 (14.3%)	0.9
**Chronic_heart_failure**	2 (13.3%)	0 (0.0%)	1 (5.3%)	2 (14.3%)	0.6
**Pulmonary_hypertension**	0 (0.0%)	0 (0.0%)	1 (5.3%)	2 (14.3%)	0.4
**Hypercholesterolemia**	2 (13.3%)	2 (25.0%)	2 (10.5%)	4 (28.6%)	0.5
**Diabetes**	1 (6.7%)	1 (12.5%)	2 (10.5%)	3 (21.4%)	0.8
**Liver_disease**	1 (6.7%)	0 (0.0%)	1 (5.3%)	2 (14.3%)	0.7
**Chirrosis**	0 (0.0%)	0 (0.0%)	1 (5.3%)	0 (0.0%)	>0.9
**Chronci_renal_failure**	0 (0.0%)	0 (0.0%)	1 (5.3%)	0 (0.0%)	>0.9
**Neurological_disease**	1 (6.7%)	0 (0.0%)	1 (5.3%)	2 (14.3%)	0.7
**Dementia**	0 (0.0%)	0 (0.0%)	0 (0.0%)	1 (7.1%)	0.4
**Rheumatological Disease**	0 (0.0%)	3 (37.5%)	0 (0.0%)	5 (35.7%)	<0.001
**Rheumatological Arthritis**	0 (0.0%)	3 (37.5%)	0 (0.0%)	2 (14.3%)	0.004
**Vasculitis**	0 (0.0%)	1 (12.5%)	0 (0.0%)	1 (7.1%)	0.2
**Osteoporosis**	1 (6.7%)	2 (25.0%)	2 (10.5%)	4 (28.6%)	0.3
**GERD**	3 (20.0%)	4 (50.0%)	5 (26.3%)	10 (71.4%)	0.019
**Ulcerative_colitis**	0 (0.0%)	2 (28.6%)	0 (0.0%)	0 (0.0%)	0.014
**Immunodeficit**	0 (0.0%)	1 (12.5%)	0 (0.0%)	0 (0.0%)	0.14
**Tumor**	3 (20.0%)	3 (37.5%)	3 (15.8%)	2 (14.3%)	0.7
**Solid_tumor**	3 (20.0%)	3 (37.5%)	3 (15.8%)	2 (14.3%)	0.7
(B)					
**Variable**	**1 N = 15 ^1^**	**2 N = 8 ^1^**	**3 N = 19 ^1^**	**4 N = 14 ^1^**	***p*-Value ^2^**
**Statins**	2 (13.3%)	2 (25.0%)	1 (5.6%)	4 (28.6%)	0.3
**Ace_inhibitors**	0 (0.0%)	0 (0.0%)	2 (11.1%)	5 (35.7%)	0.022
**Sartans**	4 (26.7%)	1 (12.5%)	6 (35.3%)	3 (21.4%)	0.7
**Aspirin**	1 (6.7%)	0 (0.0%)	0 (0.0%)	0 (0.0%)	0.7
**Clopidogrel**	2 (13.3%)	0 (0.0%)	0 (0.0%)	0 (0.0%)	0.2
**Anticoagulant_therapy**	1 (6.7%)	1 (12.5%)	1 (5.6%)	3 (21.4%)	0.5
**B_blockers**	4 (26.7%)	0 (0.0%)	4 (22.2%)	8 (57.1%)	0.031
**PPI**	6 (40.0%)	4 (50.0%)	6 (33.3%)	12 (85.7%)	0.018
**Vaccination_influenzae**	8 (57.1%)	5 (83.3%)	8 (72.7%)	8 (66.7%)	0.8
**Vaccination_antipneumoccal**	10 (71.4%)	4 (66.7%)	9 (75.0%)	8 (66.7%)	>0.9
**Anti SARS-CoV-2**					>0.9
0	1 (7.7%)	0 (0.0%)	0 (0.0%)	1 (11.1%)	
1	1 (7.7%)	0 (0.0%)	1 (9.1%)	1 (11.1%)	
2	11 (84.6%)	7 (100.0%)	10 (90.9%)	7 (77.8%)	
**Cough**	11 (73.3%)	4 (50.0%)	13 (72.2%)	12 (85.7%)	0.4
**Sputum**	13 (86.7%)	6 (75.0%)	14 (77.8%)	13 (92.9%)	0.6
**Sputum_daily**	9 (60.0%)	4 (50.0%)	9 (50.0%)	13 (92.9%)	0.047
**Haemoptysis**	5 (33.3%)	3 (37.5%)	5 (27.8%)	5 (35.7%)	>0.9
**frequency_haemoptysis**					0.7
daily	0 (0.0%)	0 (0.0%)	0 (0.0%)	1 (20.0%)	
periodic	2 (40.0%)	1 (33.3%)	3 (50.0%)	3 (60.0%)	
previous	3 (60.0%)	2 (66.7%)	3 (50.0%)	1 (20.0%)	
**Dyspnea**	6 (40.0%)	3 (37.5%)	7 (36.8%)	11 (78.6%)	0.077
**Weight_loss**	1 (6.7%)	0 (0.0%)	3 (15.8%)	2 (14.3%)	0.7
**Astenia**	2 (13.3%)	0 (0.0%)	1 (5.3%)	5 (35.7%)	0.068
**RespiratoryFKT**	9 (64.3%)	3 (37.5%)	8 (42.1%)	9 (64.3%)	0.4
(C)					
**Variable**	**1 N = 15 ^1^**	**2 N = 8 ^1^**	**3 N = 19 ^1^**	**4 N = 14 ^1^**	***p*-Value ^2^**
**Statins**	2 (13.3%)	2 (25.0%)	1 (5.6%)	4 (28.6%)	0.3
**Ace_inhibitors**	0 (0.0%)	0 (0.0%)	2 (11.1%)	5 (35.7%)	0.022
**Sartans**	4 (26.7%)	1 (12.5%)	6 (35.3%)	3 (21.4%)	0.7
**Aspirin**	1 (6.7%)	0 (0.0%)	0 (0.0%)	0 (0.0%)	0.7
**Clopidogrel**	2 (13.3%)	0 (0.0%)	0 (0.0%)	0 (0.0%)	0.2
**Anticoagulant_therapy**	1 (6.7%)	1 (12.5%)	1 (5.6%)	3 (21.4%)	0.5
**B_blockers**	4 (26.7%)	0 (0.0%)	4 (22.2%)	8 (57.1%)	0.031
**PPI**	6 (40.0%)	4 (50.0%)	6 (33.3%)	12 (85.7%)	0.018
**Vaccination_influenzae**	8 (57.1%)	5 (83.3%)	8 (72.7%)	8 (66.7%)	0.8
**Vaccination_antipneumoccal**	10 (71.4%)	4 (66.7%)	9 (75.0%)	8 (66.7%)	>0.9
**Anti SARS-CoV-2**					>0.9
0	1 (7.7%)	0 (0.0%)	0 (0.0%)	1 (11.1%)	
1	1 (7.7%)	0 (0.0%)	1 (9.1%)	1 (11.1%)	
2	11 (84.6%)	7 (100.0%)	10 (90.9%)	7 (77.8%)	
**Cough**	11 (73.3%)	4 (50.0%)	13 (72.2%)	12 (85.7%)	0.4
**Sputum**	13 (86.7%)	6 (75.0%)	14 (77.8%)	13 (92.9%)	0.6
**Sputum_daily**	9 (60.0%)	4 (50.0%)	9 (50.0%)	13 (92.9%)	0.047
**Haemoptysis**	5 (33.3%)	3 (37.5%)	5 (27.8%)	5 (35.7%)	>0.9
**frequency_haemoptysis**					0.7
daily	0 (0.0%)	0 (0.0%)	0 (0.0%)	1 (20.0%)	
periodic	2 (40.0%)	1 (33.3%)	3 (50.0%)	3 (60.0%)	
previous	3 (60.0%)	2 (66.7%)	3 (50.0%)	1 (20.0%)	
**Dyspnea**	6 (40.0%)	3 (37.5%)	7 (36.8%)	11 (78.6%)	0.077
**Weight_loss**	1 (6.7%)	0 (0.0%)	3 (15.8%)	2 (14.3%)	0.7
**Astenia**	2 (13.3%)	0 (0.0%)	1 (5.3%)	5 (35.7%)	0.068
**RespiratoryFKT**	9 (64.3%)	3 (37.5%)	8 (42.1%)	9 (64.3%)	0.4

^1^ Median (Q1, Q3); n (%). ^2^ Kruskal-Wallis rank sum test; Fisher’s exact test.

## Data Availability

Dataset available on request from the authors. The raw data supporting the conclusions of this article will be made available by the authors on request.
